# P100 Validation of cefepime/enmetazobactam on Bruker AST devices

**DOI:** 10.1093/jacamr/dlaf230.107

**Published:** 2025-12-04

**Authors:** Jon Ward, Charlotte Moter, Julia Fritz, Juan Quevedo, Esther Pfeil, Sara Klose

**Affiliations:** Advanz Pharma Medical Affairs, London, UK; MERLIN-Bruker Microbiology & Infection Diagnostics, Bornheim, Germany; MERLIN-Bruker Microbiology & Infection Diagnostics, Bornheim, Germany; Advanz Pharma Medical Affairs, London, UK; MERLIN-Bruker Microbiology & Infection Diagnostics, Bornheim, Germany; MERLIN-Bruker Microbiology & Infection Diagnostics, Bornheim, Germany

## Abstract

**Objectives:**

To evaluate the feasibility and manufacturing robustness of integrating the novel β-lactam/β-lactamase inhibitor combination cefepime/enmetazobactam (FEP/META) which has been approved by FDA, EMA and MHRA, into Bruker’s antimicrobial susceptibility testing (AST) devices. Specifically, we investigated the accuracy, stability and production compatibility of FEP/META within broth microdilution-based AST systems using both manual and automated methodologies.

**Methods:**

An initial feasibility assessment was performed using 96-well microbroth dilution plates, manually filled with FEP/META solution and vacuum-dried. Susceptibility testing was conducted using four quality control strains: *Escherichia coli* ATCC 25922, *Pseudomonas aeruginosa* ATCC 27853, *E. coli* ATCC 35218 and *Klebsiella pneumoniae* ATCC 700603. Each strain was tested in biological triplicates with variable inoculum densities. MICs were determined both visually and using two photometers. To evaluate solution stability, testing was repeated after 28 days using the same preparation. Subsequently, an automated filling process was employed to simulate production-scale manufacturing under two distinct methods (Parameter A and Parameter B). AST was conducted using the previously mentioned strains as biological triplicates with variations in inoculum density over 3 days, and MICs recorded visually and photometrically.

**Results:**

Of the 288 MIC values generated during the manual feasibility study, 100% fell within CLSI-defined quality control (QC) ranges. After 28 days of storage, 287/288 MIC results remained within QC, confirming the solution's short-term stability. The single deviation was attributed to a photometer anomaly, not compound degradation. Under automated production, 861 out of 864 MIC results (99.7%) were within QC ranges across both production parameters. All deviations were traced to a single skipped well, consistently identified across visual and photometric readings, suggesting an isolated technical anomaly with a frequency of 1 in 288 biological data points (3 out of 864 total readings). While both production settings yielded equivalent QC compliance, parameter B demonstrated more uniform MIC distribution within target ranges and was selected for future use.

**Conclusions:**

This multi-phase *in-vitro* study demonstrated that FEP/META can be effectively and reliably integrated into Bruker’s AST devices using both manual and automated processes. Across three independent experiments and 1440 total MIC readings, 99.7% fell within CLSI QC ranges, confirming analytical robustness. Additionally, FEP/META antibiotic solution showed stability over at least 28 days. Based on comparative performance, production process parameter B was chosen for continued development. These efforts will support the deployment of FEP/META in AST platforms as a reliable option for detecting susceptibility in Enterobacterales and *P. aeruginosa*.

